# Triad3A involved in the regulation of endotoxin tolerance and mycobactericidal activity through the NFκB‐nitric oxide pathway

**DOI:** 10.1002/iid3.925

**Published:** 2023-07-24

**Authors:** Yongwei Qin, Jinliang Chen, Kuang Xu, Yang Lu, Feifan Xu, Jiahai Shi

**Affiliations:** ^1^ Department of Clinical Laboratory The Sixth People's Hospital of Nantong Nantong Jiangsu China; ^2^ Department of Pathogen Biology, School of Medicine Nantong University Nantong China; ^3^ Department of Respiratory Medicine The Second Affiliated Hospital of Nantong University, Nantong First People's Hospital Nantong Jiangsu China; ^4^ Department of Critical Care Medicine Affiliated Hospital of Nantong University Nantong China; ^5^ Nantong Key Laboratory of Translational Medicine in Cardiothoracic Diseases, Nantong Clinical Medical Research Center of Cardiothoracic Disease, Institution of Translational Medicine in Cardiothoracic Diseases Affiliated Hospital of Nantong University Nantong Jiangsu China

**Keywords:** antimycobacterial activity, endotoxin tolerance, immune cell exhaustion, miRNA, Triad3A, tuberculosis sepsis

## Abstract

**Introduction:**

Sepsis is characterized by an endotoxin tolerance phenotype that occurs in the stage of infection. Persistent bacterial infection can lead to immune cell exhaustion. Triad3A, an E3 ubiquitin ligase, negatively regulates its activation by TLR4. However, the effect of Triad3A on endotoxin tolerance and bactericidal ability in the state of endotoxin tolerance remains unclear.

**Methods:**

Using single dose LPS and repeated LPS stimulated macrophage cell lines at indicated times, we investigated miR‐191, Tirad3A, TRAF3, TLR4, p‐P65, TNF‐α, IL‐1β, and iNOS expression, the effect of miR‐191 on Triad3A and TRAF3, gene loss‐of‐function analyses, the effect of Triad3A on TLR4, p‐P65, cytokine, and mycobactericidal activity in endotoxin tolerant cells infected with *Mycobacterium marinum*.

**Results:**

Here we found that Triad3A is involved in regulating endotoxin tolerance. Our result also displayed that miR‐191 expression is downregulated in macrophages in the state of endotoxin tolerance. miR‐191 can directly bind to Triad3A and TRAF3. Additionally, knockdown of Triad3A can reverse the effect of decreasing TNF‐α and IL‐1β in endotoxin tolerant macrophages. Furthermore, we demonstrated that the TLR4‐NF‐κB‐NO pathway was associated with Triad3A and responsible for the killing of intracellular mycobacteria in a tuberculosis sepsis model.

**Conclusions:**

These results provide new insight into the mechanisms of Triad3A induced tolerogenic phenotype in macrophages, which can help the better comprehension of the pathogenesis involved in septic shock with infection of *Mycobacterium* *tuberculosis*, and suggest that Triad3A may be a potential drug target for the treatment of severe septic tuberculosis.

## Graphical abstract

Triad3A regulated endotoxin tolerance and mycobactericidal activity. miR‐191 expression is downregulated in endotoxin‐tolerant macrophages and mediates upregulation of Triad3A and TRAF3 expression, which can further inhibit the expression of TLR4. The expression of Triad3A and TRAF3 is increased in *Mycobacterium marinum* infected macrophages, Triad3A can degrade TLR4, and TRAF3 negatively regulates NF‐κb, then synergically decrease the NF‐κB activity. Thereby, the expression of iNOS and NO is decreased. The downregulation of NO results in the accumulation of *M. marinum* in the macrophage.

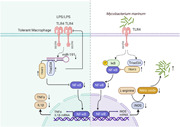

## 1 INTRODUCTION

Endotoxin tolerance (ET), a phenomenon, observed both in vivo and in vitro in humans as well as in animal models,^1^, ^2^, ^3^ in which organisms or cells exposed to low concentrations of endotoxin (e.g., Lipopolysaccharides [LPS]) enter into a transient tolerant state and are incapable of responding to further challenges with endotoxin. It is postulated that endotoxin tolerance, serving as a classic protective mechanism, played a role in regulating over‐exuberant inflammation for individual protection against endotoxin shock.^4^ Importantly, the occurrence of ET has been reported in a series of diseases, including sepsis, surgery, pancreatitis, and trauma, highlighting its clinical implications.^1^ However, the molecular mechanism of ET remains unclear although recent progress has suggested its role in multilayer modulation of inflammation,^5^ including Toll‐like receptor signaling^6^ and the identification of patent molecules through systems biology as well as genetic dissection.^7^, ^8^ Sepsis is one of the most common clinical ET‐associated diseases. It has been reported that *Mycobacterium tuberculosis* infection was involved in septic shock with multiple organ dysfunctions in the immunocompetent host.^9^, ^10^, ^11^, ^12^, ^13^, ^14^, ^15^ Survival of tuberculosis patients in intensive care units (ICU) has not ameliorated, and mortality is still high.^16^ Similarly, the potential molecular mechanism of tuberculosis sepsis was less well known.

Tuberculosis (TB) is one of the most severe public health problems worldwide. One‐third of the world's population has been infected with TB, with 1.2 million deaths per year (Global Tuberculosis Report, WHO, 2020). Macrophages are key host‐defense cells against *M. tuberculosis* and are critical for the pathogenesis of tuberculosis. Macrophages attempt to eradicate *M. tuberculosis* by several microbicidal mechanisms, including antimicrobial peptides, lysosomal degradation, reactive oxygen/nitrogen species, cytokine‐mediated mechanisms, recruitment of immune cells, and activation of adaptive immunity by presenting antigens to T cells.^17^ However, multiple levels of negative regulators are manipulated by *M. tuberculosis* to effectively subvert the bactericidal mechanisms to facilitate its proliferation in macrophages.^18^, ^19^

Although mycobacteria have developed strategies to subvert host defense, pathogen‐associated molecular patterns of mycobacteria, such as lipoglycans and lipoproteins, are recognized through the Toll‐like receptors (TLRs) in the immune cells, especially TLR2 and TLR4.^20^ TLRs played a decisive role in the fight against Mycobacteria. TLRs initiated rapid defense responses such as activation of microbicidal activity and phagocytosis,^21^ and regulated oncoming acquired immune responses.^22^ Nevertheless, TLRs may also be objected by mycobacteria in their attempt to escape from host immune eradication. Finally, studies have deciphered how mycobacteria destroy TLRs to escape from host immune killing. A study showed that LPS can facilitate *M. tuberculosis* localization to autophagosomes in macrophages infected with mycobacteria, emphasizing the function of TLR4 in mediating the autophagy pathway, which may be targeted by *M. tuberculosis* to increase its intracellular survival.^23^

Triad3A, a RING finger E3 ligase, mediates the ligation of Ubiquitin to TLR4 and TLR9, resulting in protein degradation and negative modulation of TLR signaling.^24^ It has been reported that the expression of TLR signaling cascades is downregulated in endotoxin‐tolerant mononuclear cells and macrophages.^25^, ^26^ TLR signaling pathways can be harnessed in infected and inflammatory diseases. Therefore, we reasoned that Triad3A modulated proteolytic degradation of TLRs with a concomitant decrease in signaling. In this study, we proved that the activity of Triad3A was regulated or harnessed by mycobacteria, which thereby reduced TLR signaling.

miRNAs (miRs) can modulate gene expression at the transcriptional level, there are mounting studies demonstrating that miRs, such as miR‐146a, miR‐221, miR‐579, miR‐125b, miR‐155, let‐7e, and miR‐98, are involved in regulating the TLR4 signaling pathway during the development of endotoxin tolerance.^27^, ^28^, ^29^ miR‐191 has been reported to regulate cell differentiation, proliferation, apoptosis, and migration by targeting key transcription factors. It is also involved in several other diseases, such as neurodegenerative diseases, type 2 diabetes, Crohn's disease, pulmonary hypertension, and Aneurysmal Aortic Remodeling.^30^, ^31^ Studies have shown that miR‐191 is highly expressed in LPS‐treated PBMCs and plays an important role in the immune response.^32^ These findings highlighted miR‐191's role in the regulation of various disorders.

*Mycobacterium marinum*, a relatively new model organism for the study of mycobacterial tuberculosis pathogenesis, is regarded as reliable for application under BSL‐2 laboratory conditions, making it a widely applicable organism. *M. marinum* grows colonies on agar for 7–9 days, while *M. tuberculosis* takes 3 weeks. The pathological similarity of *M. tuberculosis* to *M. marinum* infection, coupled with its improved safety profile, faster growth, and ease to use, makes *M. marinum* a superior model for mycobacterial pathogenic research.^33^, ^34^

Due to the widespread function of Triad3A and miR‐191 in TLR signaling and inflammation, we postulated that miR‐191 was involved in regulating the ET by targeting Triad3A. Although ET has been extensively studied in human monocytes and murine macrophages, the effect of ET on bacterial infection and the bactericidal activity of macrophages remains elusive. It is still unknown whether the bactericidal activities of macrophages are promoted or not in tuberculosis sepsis. Here we found Triad3A, along with miR‐191, and participate in regulating ET. Knockdown of Triad3A or overexpression of miR‐191 could significantly affect the state of inflammation and ET in the *M. marinum*‐infected macrophages. Our results provide new insights to understand the regulating mechanism of ET and the role of miR and Triad3A in maintaining macrophage inflammation and the activity of bacterial clear.

## 2 MATERIALS AND METHODS

### 2.1 Cell culture and treatment

RAW 264.7, BV2, and HEK 293 cell lines were purchased from the American Type Culture Collection (ATCC). Cells were maintained in DMEM (Gibco) medium supplemented with 10% fetal bovine serum, 100 U/mL penicillin and 100 mg/mL streptomycin (Gibco) in the humidified chamber at 37°C with 5% CO_2_.

Primary mouse peritoneal macrophages (MPM) were separated from C57BL/6J mice (*n* = 12). Two milliliters of 4% thioglycollate was intraperitoneally injected in mice. After 3 days, peritoneal exudate cells were separated by lavaging the peritoneal cavity using Hank's Balanced Salt Solution. These cells were cultured for 8 h, and adherent cells were used as peritoneal macrophages. Glial cultures were prepared from the cerebral cortices of 1‐day‐old C57BL/6 mice as previously described by Saura et al.^35^ Briefly, the cortex was quickly dissociated and plated in culture flasks. Two weeks later, the mixed glial cultures were trypsinized using 0.05% trypsin with 0.25 mM EDTA in DMED‐F12 for 20 min, which obtain a layer of loosely appendiculate astrocyte and leaves behind tightly adherent cells, >95% of which are microglia. The experimental protocol was approved by the Animal Care Committee of Nantong University.

### 2.2 Cell tolerance model

To induce endotoxin tolerance, cells were pretreated with 10 ng/mL LPS (Sigma‐Aldrich; Merck KGaA) for 18 h, followed by washing LPS with PBS. Cells were rested for 2 h before retreatment with 100 ng/mL LPS for 0, 6, or 12 h, as described previously.^3^, ^4^, ^36^ To induce inflammation, cells were directly stimulated with 100 ng/mL LPS. RNA, protein, and cell‐free supernatants were collected at the indicated times, following the ultimate stimulation, respectively.

### 2.3 Bacterial strains and culture

*M. marinum* (ATCC) was cultured in Middlebrook 7H9 broth (Difco) with 10% oleic acid‐albumin‐dextrose‐catalase (BD Biosciences, NZ, USA), 0.5% glycerol and 0.05% Tween‐80 in a 28°C–32°C incubator. Intracellular survival assays were described previously.^37^, ^38^

### 2.4 Bactericidal activity assay

The infected cells were washed thrice with PBS and then lysed cell membranes with 0.1% Triton X‐100 to release intracellular mycobacterium. The number of intracellular bacteria (CFU) was counted by plating appropriate dilutions on the 7H10 agar plates.

### 2.5 Generation of Triad3A knock‐down cells

shRNA Triad3A and shRNA Scramble were transfected into RAW 264.7 by using the jetPRIME® transfection reagent (Polyplus‐transfection Inc) according to the manufacture's protocol (https://www.polyplus-transfection.com/blog/jetprime-protocol-video/). The stable Cells were selected beginning at 48 h posttransfection for 3 weeks in DMEM containing 10% FBS, and 10 μg/mL puromycin (Invitrogen).

### 2.6 miRNA mimic and inhibitor transfection

Raw 264.7 and BV2 cells were incubated in 6‐well plates and transfected with miRNA mimic and inhibitor when the cell density reached approximately 80% confluency. miR‐191 mimic, miR‐191 inhibitor and corresponding Negative Control (NC) were transfected using jetPRIME® transfection reagent according to the manufacturer's instructions. After 48 h, cells were treated with Medium/LPS or LPS/LPS for indicated times.

### 2.7 Immunoblotting

Cell lysates were used to SDS‐PAGE, then transferred to polyvinylidene difluoride membranes. Target proteins were detected with primary antibodies against Triad3A (ab25961; Abcam), TRAF3, TLR4 (sc‐1828, sc‐293072; Santa Cruz Biotechnology), phosphorylated‐NF‐κB P65 (#3033; Cell Signaling Technology), iNOS (ab49999; Abcam), β‐Actin Mouse Monoclonal Antibody, HRP‐conjugated antirabbit or antimouse secondary antibodies (TransGen). Bands were visualized using ECL (Millipore). Bands densitometry was quantified by ImageJ software (National Institutes of Health).

### 2.8 ELISA

Supernatants were collected and the expression of cytokines was assayed using the BD optEIA^TM^ ELISA kit (BD Bioscience) according to the manufacturer's protocols. The absorbance value was read at 450–570 nm using a microplate reader (Multiskan EX; Thermo Scientific).

### 2.9 Real‐time PCR

RNAs were extracted using the RNeasy Mini Kit. First‐strand cDNAs were synthesized using Reverse Transcriptase (Qiagen). Quantitative PCRs (qPCRs) were performed in a CFX96^TM^ Real‐Time System (Bio‐Rad), using SYBR green PCR mixture kits (Takara). miRNA qPCR Primers were used to determine miRNA expression levels. RNU6‐1 was used for miRNA template normalization. GAPDH was used as for gene expression endogenous control. Relative quantity of transcripts was calculated using the $2^{-\Delta \Delta C_{t}}$ formula. All PCR primers are listed in Table 1. All data are presented as relative quantification.

**Table 1:** Primer used in this study.

Gene	Sequences (5′−3′)
Triad3A 3′UTR	Forward: CCGCTCGAGTCCGTGGAAAGTCAGGTTGAAAGTAT
	Revese: ATAAGAATGCGGCCGCCAAGGACAGCCAGTACAACTGAGAAA
Triad3A 3′UTR‐mut	Forward: ATGACCTCATGGAGCTGCTACAGT
	Reverse: GTCCTCAACAACCCCCAAGGGACCG
Triad3A shRNA1	Forward: gatccTCTTTGCAGAACAAGTGAGTTCAAGAGACTCACTTGTTCTGCAAAGAtttttg
	Reverse: aattcaaaaaTCTTTGCAGAACAAGTGAGTCTCTTGAACTCACTTGTTCTGCAAAGAg
Triad3A shRNA2	Forward: gatccGAGCAGGAGTTCTATGAGCTTCAAGAGAGCTCATAGAACTCCTGCTCtttttg
	Reverse: aattcaaaaaGAGCAGGAGTTCTATGAGCTCTCTTGAAGCTCATAGAACTCCTGCTCg
Scramble shRNA	Forward: gatccTTCTCCGAACGTGTCACGTTTCAAGAGAACGTGACACGTTCGGAGAAtttttg
	Reverse: aattcaaaaaTTCTCCGAACGTGTCACGTTCTCTTGAAACGTGACACGTTCGGAGAAg
TRAF3 3'UTR2	Forward: CCGCTCGAGGTGTGCAAACCACCACATCTGTGCC
	Revese: ATAAGAATGCGGCCGCTACAGGAAGCTGTGACATTGTTC
TRAF3 3′UTR3	Forward: CCGCTCGAGGTCTGGAGTCTGTCCTGTGAGCTC
	Revese: ATAAGAATGCGGCCGCCCTGGTGACATGATTATTGCTGAGC
TRAF3 3′UTR mut1	Forward: GGCCCGCCCTCTCAGCTCACTGTTCC
	Revese: TCAGGAGTTCCTCCCGTCGGTCGACTACGGG
TRAF3 3′UTR mut2	Forward: AATCAACAGGTGTAGGCCAGCAG
	Revese: TCAGGAGTCATCGAGGGGGGAAGTCTCTTTAG
TRAF3 3′UTR mut3	Forward: CAGCTGCAGACTGTTCTAAAGTTCCGT
	Revese: TCAGGAGTACCCGCGAGACTGACTCATCCAACCCCA
mmu‐miR‐191	RT: GTCGTATCCAGTGCAGGGTCCGAGGTATTCGCACTGGATACGACGGGAAC
	Forward: CCCGTCGTATCCAGTGCGAATACCT
	Revese: CAGTGCAGGGTCCGAGGTAT
mmu‐miR‐191 mimic	GCUGCACUUGGAUUUCGUUCCC (double strand)
mmu‐miR‐191 inhibitor	GGGAACGAAAUCCAAGUGCAGC
Mimic negative control	UUCUCCGAACGUGUCACGU (double strand)
Inhibitor negative control	ACGUGACACGUUCGGAGAA
RNU6‐1	RT: AACGCTTCACGAATTTGCGT
	Forward: CTCGCTTCGGCAGCACA
	Revese: AACGCTTCACGAATTTGCGT
TNF‐α	Forward: GCTGAGCTCAAACCCTGGTA
	Revese: GCTGAGCTCAAACCCTGGTA
IL‐1β	Forward: GGATGAGGACATGAGCACCT
	Revese: AGCTCATATGGGTCCGACAG
GAPDH	Forward: TGACCTCAACTACATGGTCTACA
	Revese: CTTCCCATTCTCGGCCTTG

### 2.10 3′ UTR luciferase reporter assays

The 3′ UTR of mouse Triad3A and TRAF3 were amplified by PCR from RAW 264.7 and cloned downstream of the Renilla luciferase sequence, between the *Xho* I and *Not* I site of the psiCHECK2 reporter vector (Promega). The miR‐191 target sites in the Triad3A and TRAF3 3′ UTR were mutated by mutating the 3–4 nt using the Site‐Directed Mutagenesis Kit (Stratagene) according to the manufacturer's instructions. HEK 293 cells were cotransfected with 100 ng of psiCHECK2‐Triad3A 3′ UTR or psiCHECK2‐Triad3A 3′ UTR‐mut, psiCHECK2‐TRAF3 3′ UTR or psiCHECK2‐TRAF3 3′ UTR‐mut luciferase plasmid, and the indicated miR‐191 or NC (Negative Control) (final concentration, 50 nM) (Ribio Biotech) using Lipofectamine 2000. After 24 h, luciferase activities were assayed using the Dual‐Glo Luciferase Reporter System (Promega). Data were normalized by dividing Renilla luciferase activity with that of firefly luciferase.

### 2.11 NO assay (Griess test)

The concentrations of nitrate in the culture supernatants harvested from the culture cells were detected by adding 50 μL of Griess reagent (Promega) to 50 μL of the sample in a 96‐well plate, and then the absorbance was read at 540 nm in the dark.

### 2.12 Flow cytometry assay

The expression of macrophage surface receptor, TLR4 was determined on RAW 264.7 cells infected with *M. marinum* for the indicated times. Cells were harvested from 24‐well plates, and incubated with Fc‐blocking reagents first, then incubated with antimouse PE‐TLR4 (145404; BioLegend) for 30 min at 4°C, protected from light. PE Rat IgG2a, κ Isotype Control (BioLegend) was used. The cells were washed with phosphate‐buffered saline (1%BSA), and the Flow Cytometry was used to measure the TLR4 expression. The data were analyzed by FlowJo software.

### 2.13 Statistical analyses

Experiments were assayed at least three times. Statistical analysis was performed using Prism 7.0 GraphPad Software. The difference between two groups was analyzed by two‐tailed Student's *t* test. One‐way analysis of variance followed by Tukey's post hoc test was used to compare multiple groups. Two‐way ANOVA was used to compare the indicated times of inflammatory groups and endotoxin tolerance groups. All data were displayed as mean ± SD. A *p* < 0.05 was considered significant.

## 3 RESULTS

### 3.1 The effects of inflammation and endotoxin tolerance on Triad3A expression in macrophages

**Figure 1:**
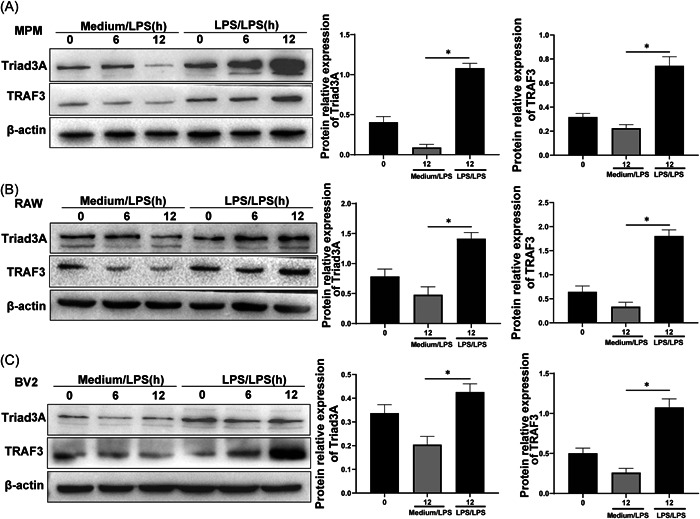
Triad3A is involved in endotoxin tolerance. To induce inflammation (Medium/LPS) and ET (LPS/LPS), cells were treated with a single injection of 100 ng/mL LPS, or 10 (low dose)−100 (high dose) ng/ml LPS, respectively. Protein expression was detected in the MPM (A), RAW 264.7 (B) and BV2 (C) using Western blot. ET, endotoxin tolerance; LPS, Lipopolysaccharides; MPM, mouse peritoneal macrophages.

Endotoxin tolerance is regarded as a reduced capacity of the host or of cultured macrophages to respond to LPS activation. LPS has the property of inducing endotoxin tolerance to its own biological effects.^39^ This phenomenon has been extensively studied in animal models, but its mechanism has not been completely clarified. Here we investigate whether Triad3A is implicated in regulating endotoxin tolerance in macrophages during the induction of LPS tolerance by repeated LPS treatment. Macrophages and microglia are classic innate immune cells. We then detected the expression of Triad3A in mouse peritoneal macrophage (MPM) (Figure 1A), murine macrophage RAW 264.7 (Figure 1B), and murine microglia BV2 cells (Figure 1C). Cells were treated with medium/LPS (inflammation group) or LPS/LPS (endotoxin tolerance group), respectively. We found the levels of Triad3A in endotoxin tolerance cells were significantly increased after treatment with LPS/LPS (Figure 1A–C). TRAF3, an E3 ubiquitin ligase, can facilitate the polyubiquitination of pro‐inflammatory mediators.^40^ Studies have shown that Triad3A downregulates the RIG‐I‐like receptor pathway by proteasomal degradation of TRAF3 through the Lys48‐linked ubiquitin degradation pathway.^41^ The above results encouraged us to analyze TRAF3 on inflammatory or endotoxin tolerance cells that might synergize with Triad3A for mediating endotoxin tolerance. Next, we detected the level of TRAF3 using immunoblotting. We also found that the level of TRAF3 was upregulated in endotoxin tolerance MPM, RAW 264.7, and BV2 compared with control cells treated with medium/LPS (Figure 1A–C).

### 3.2 miR‐191 negatively regulates Triad3A and TRAF3

**Figure 2:**
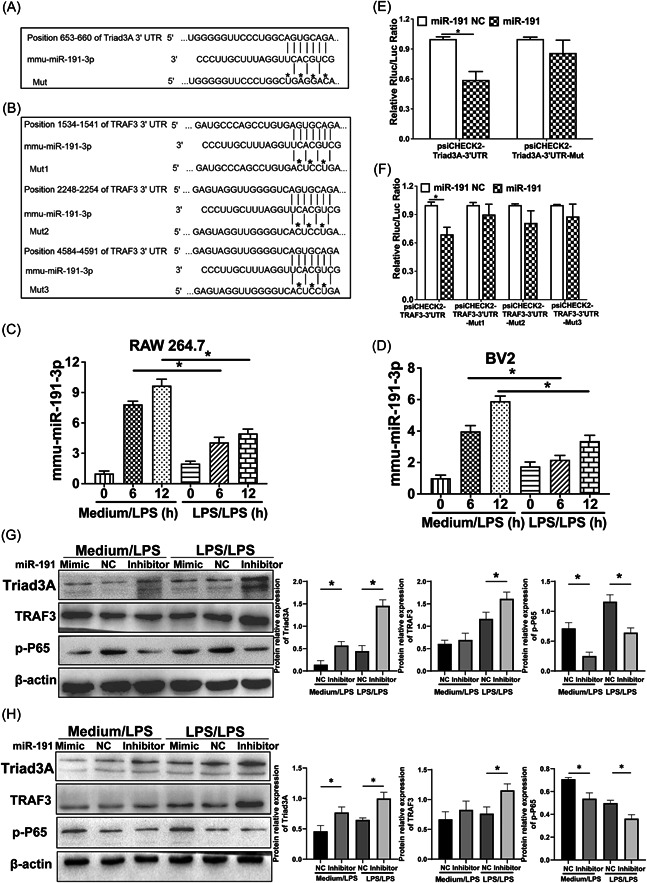
Translational inhibition of Triad3A by miR‐191. (A and B) Sequence alignment between miR‐191 and its theoretical binding sites in the 3′ UTR of Triad3A and TRAF3 mRNA. The sequence of Triad3A and TRAF3 3′ UTR mutants used for reporter assay is also shown (asterisk); (C and D) Expression of miR‐191 in the RAW 264.7 and BV2 cells. Experiments were performed at least three biologically independent replicates and data are presented as the mean ± SD. (E and F) Luciferase reporter constructs plasmid containing 3′ UTR (psiCHECK2‐Triad3A or TRAF3 3′ UTR) or mutant 3′ UTR (psiCHECK2‐Triad3A or TRAF3 3′ UTR‐mut) were transfected with miR‐191 or miR‐191 NC in HEK 293 cell and the luciferase activities (Renilla/Firefly) were assayed; (G and H) the effect of miR‐191 on Triad3A, TRAF3 and phosphorylated P65 (p‐P65) were detected in RAW 264.7 (G) and BV2 cells (H) transfected with miR‐191 mimic and inhibitor.

miR‐191 has previously been reported to be expressed in inflammation and cancer.^30^, ^32^ Consistently, we speculated if the silent expression of miR‐191 was implicated in endotoxin tolerance. We performed the potential target searching using TargetScan (http://www.targetscan.org) and showed that miR‐191 could directly target Triad3A and TRAF3 by binding to their 3'‐UTR regions (Figure 2A,B). To further investigate whether miR‐191 participates in endotoxin tolerance through Triad3A or TRAF3. First, RAW 264.7 and BV2 were treated with LPS to induce inflammation or repeated LPS to induce endotoxin tolerance. The results displayed that miR‑191 levels were gradually upregulated after LPS treatment for 6 and 12 h (Figure 2C,D). Following treatment with repeated LPS, upregulation of miR‑191 was not more drastic compared with the inflammatory group. Another study revealed that miR‐191 was significantly upregulated (5.13‐fold change) in tuberculosis patient.^42^ These results indicate that miR‐191 participates in endotoxin tolerance and inflammation.

To confirm the effect of miR‐191 on Triad3A and TRAF3 expression through direct interaction with their 3'UTR, we cloned 3′ UTR or 3′ UTR‐mut of Triad3A and TRAF3 into psiCHECK2 luciferase reporter vector and performed a luciferase reporter analysis in HEK 293 cells. Our results displayed that cotransfection of miR‐191 mimics with Triad3A and TRAF3 3′ UTR reporter, resulting in inhibition of luciferase activity. However, miR‐191 failed to inhibit the activity of mutated Triad3A and TRAF3 3′ UTR reporters (Figure 2E,F), indicating a direct binding between miR‐191 and 3′ UTR regions. To verify whether miR‐191 can modulate Triad3A and TRAF3 expression in inflammatory or tolerant RAW 264.7 or BV2 cells, we examined the endogenous Triad3A, TRAF3 and their downstream protein, NF‐κB. Overexpression of miR‐191 (mimic) results in a potent downregulation of Triad3A and TRAF3 levels, whereas concomitant transfection with anti‐miR‐191 (inhibitor) efficiently antagonized the miR‐191‐mediated Triad3A inhibition compared to the anti‐miR‐191 NC (negative control). Triad3A prevents LPS‐induced NF‐κB p65 activation by catalyzing K48 polyubiquitination of TLR4.^43^ Interestingly, the level of p‐P65 was the very opposite trend of the expression of Triad3A and TRAF3 proteins in RAW 264.7 and BV2 (Figure 2G,H). These data indicate that Triad3A and TRAF3 are direct targets of miR‐191 in inflammatory and endotoxin tolerant macrophages. NF‐κB p65 may participate in the regulation of endotoxin tolerance by miR‐191.

### 3.3 Stable knockdown of Triad3A correlates with promoted NF‐κB P65 and pro‐inflammatory cytokine expression in tolerant macrophages

**Figure 3:**
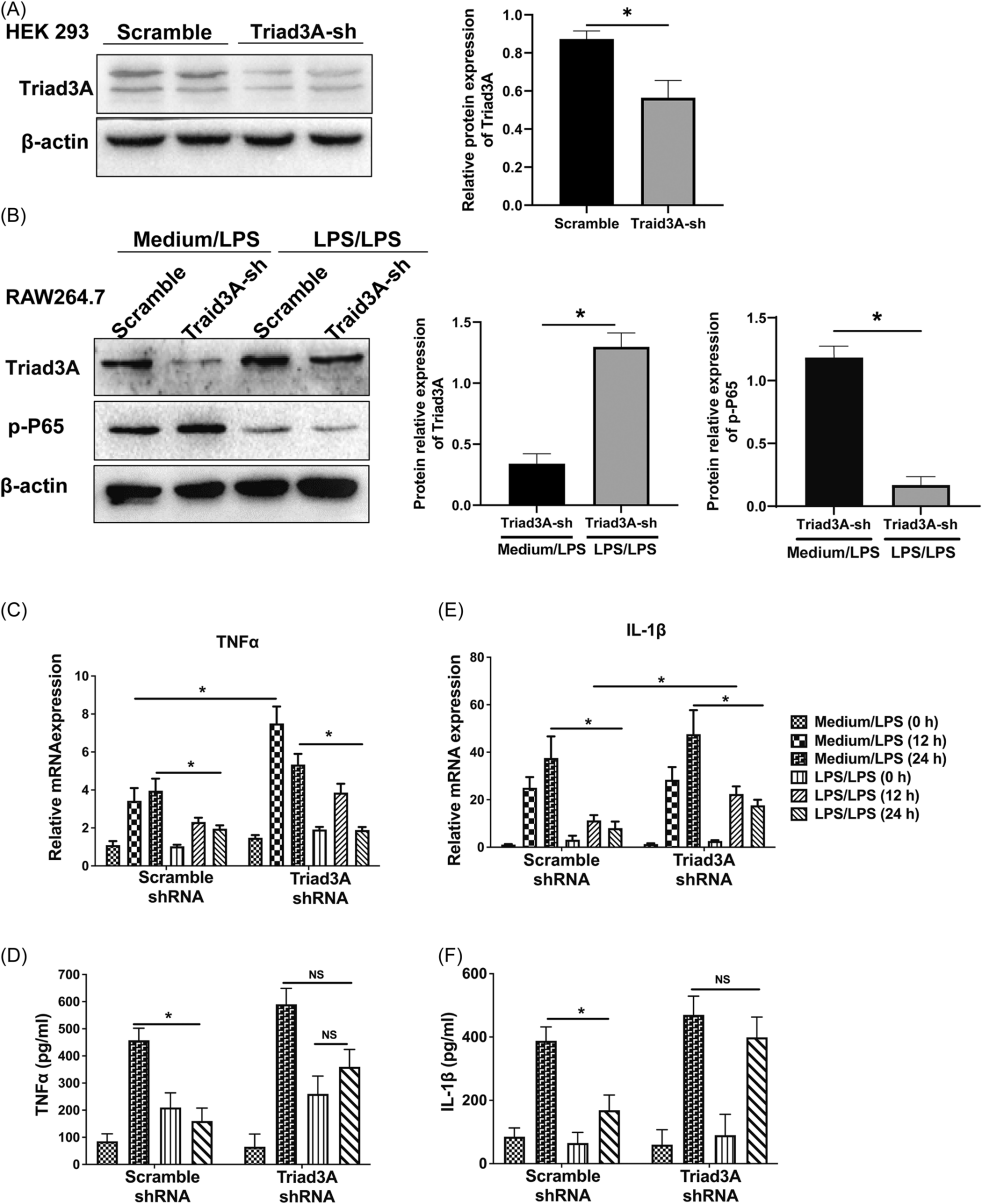
Triad3A is involved in the induction of ET in the macrophage. (A) To measure the shRNA1 and shRNA2‐mediated knockdown of Triad3A expression in HEK 293 cells. (B) RAW 264.7 cells transfected with either scramble shRNA, or Triad3A shRNA were subsequently treated with 100 ng/mL LPS to induce inflammation or repeated 10–100 ng/mL LPS to induce ET. Following 24 h, Triad3A and phosphorylated P65 were detected by western blot. (C–F) mRNA and protein levels of TNF‐α (C and D) and IL‐1β (E and F) were measured using qPCR and enzyme‐linked immunosorbent assays, respectively. All data are presented as the mean ± SD of three independent experiments. Two‐way ANOVA was used for the statistical analysis. *p < .05. ET, endotoxin tolerance; LPS, lipopolysaccharides.

Reports have suggested that Triad3A mediates the ligation of ubiquitin to TLR4 and TLR9, leading to protein degradation and negative modulation of TLR signaling.^24^ Previous studies also demonstrated that NF‐κB and AP‐1 take part in regulating LPS tolerance in the human monocytic cell.^44^ To further identify whether the effect of miR‐191 on modulating endotoxin tolerance is exerted through Triad3A‐NF‐κB P65, we knocked down Triad3A using short hairpin RNAs (shRNAs) specific for Triad3A and examined the effect of Triad3A‐knockdown on the expression of NF‐κB P65 and pro‐inflammatory cytokines, including TNF‐α and IL‐1β during single or repeated LPS exposure. First, HEK 293 cells were transfected with either pGreenPuro‐shRNA‐Triad3A (Triad3A‐Knockdown) or pGreenPuro‐scramble (Triad3A‐Scramble) and the knockdown efficiency was confirmed by western blot (Figure 3A). The result displayed that expression of Triad3A was decreased in cells transfected with Triad3A shRNA compared with those transfected with scramble shRNA. It was demonstrated that upregulation of Triad3A elicited by LPS/LPS was counteracted by Triad3A shRNA compared with those transfected with pGreenPuro‐shRNA‐Scramble (Figure 3B). Meanwhile, LPS/LPS significantly downregulated p‐P65 expression in cells stably transfected either with Triad3A shRNA‐scramble or with those transfected with pGreenPuro‐shRNA‐Triad3A (Figure 3B). Next, we examined the expression levels of inflammatory cytokines. In cells treated with LPS/LPS for 12 h to induce endotoxin tolerance, the expression of TNF‐α and IL‐1β mRNA was significantly decreased compared to that in unprimed cells (Figure 3C,E). Notably, the expression of both TNF‐α and IL‐1β could be rescued by silencing the expression of Triad3A. In cells transfected with Triad3A shRNA to induce Triad3A knockdown, medium/LPS significantly increased TNF‐α and IL‐1β mRNA production compared with those treated with LPS/LPS (Figure 3C,E). Unexpectedly, in cells transfected with Triad3A shRNA, the secretion of TNF‐α and IL‐1β showed no significant difference between medium/LPS treated cells and LPS/LPS treated cells, partially due to the counteraction of shRNA (Figure 3D,F). Taken together, these results indicated that Triad3A played a critical role in endotoxin tolerance, probably by downregulating p‐P65 and pro‐inflammatory cytokines TNF‐α and IL‐1β.

### 3.4 The effect of *M. marinum* on Triad3A, TRAF3, and TLR4 expression, stable knockdown of Triad3A correlates with promoted nitric oxide (NO) expression following *M. marinum* infection

**Figure 4:**
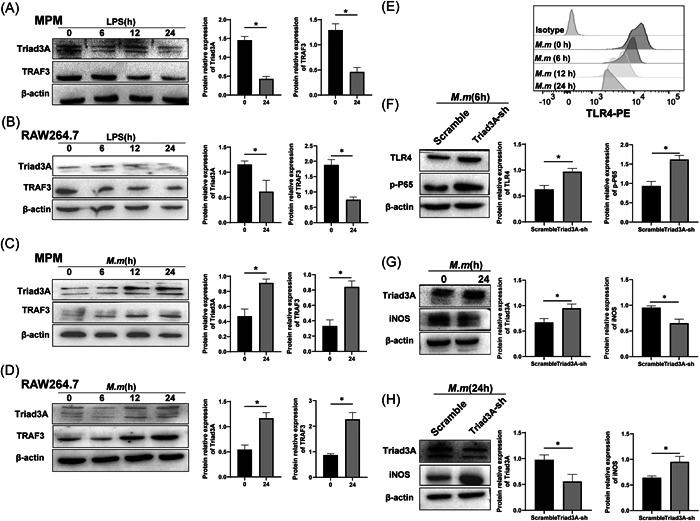
Triad3A is induced by *Mycobacterium marinum* and regulates the TLR4‐NF‐κB pathway. (A and B) MPM and RAW 264.7 were treated with 100 ng/mL LPS for 6, 12, or 24 h. (C and D) MPM and RAW 264.7 were infected with *M. marinum* at an MOI of 1 for 6, 12, or 24 h. Triad3A and TRAF3 expression were detected using Western blot. (E) The expression of TLR4 levels was measured using Flow Cytometry. (F–H) Stable transfection of Triad3A shRNA and Triad3A Scramble RAW 264.7 were infected with *M. marinum* at an MOI of 1 for 24 h. p‐P65, Triad3A, and iNOS expression were detected using Western blot, respectively. MPM, mouse peritoneal macrophages.

Previous studies demonstrated that Triad3A or TRAF3 participates in regulating the signal pathway of inflammation ^45^ and RNA virus infection.^41^ We hypothesized that Triad3A or TRAF3 could play a role in controlling the bacterial infection through the TLR‐NO pathway, thus we detected the expression of Triad3A and TRAF3, and the change of signal pathway caused by Triad3A in cells infected with *M. marinum*. The expression of Triad3A and TRAF3 was markedly decreased in LPS‐treated RAW 264.7 and MPM cells (Figure 4A,B), whereas the expression of Triad3A and TRAF3 was upregulated in cells infected with *M. marinum* (Figure 4C,D). The previous report showed that viable *M. tuberculosis* bacilli contain distinct ligands that activate cells via TLR2 and TLR4.^22^ Other reports showed that the expression of TLR was changed by LPS treatment.^46^ We herein demonstrated that expression of TLR4 protein was downregulated in *M. marinum* infected RAW 264.7 (Figure 4E). To confirm the effects of degradation of TLR4 by Triad3A in *M. marinum* infected RAW 264.7, we transfected Triad3A‐shRNA into RAW 264.7 infected with *M. marinum* and found that TLR4 and p‐P65 expression significantly increased with downregulation of Triad3A (Figure 4F), suggesting that *M. marinum* downregulates TLR4 level at least partially depending on Triad3A. As mentioned above, we found Triad3A could down‐regulate the NF‐κB pathway. Previous studies also demonstrated that NF‐κB participates in inducible nitric oxide synthase (iNOS) gene regulation^47^, ^48^ and iNOS exerts potent intracellular mycobactericidal activity.^49^, ^50^ We investigated whether Triad3A is associated with the antimycobacterial activity of macrophages by NO. We first found that the upregulation of Triad3A was associated with decreased iNOS levels in RAW 264.7 infected cells treated with *M. marinum* for 24 h (Figure 4G). To clarify the effects of Triad3A on iNOS, we similarly transfected Triad3A‐shRNA into RAW 264.7 infected with *M. marinum* and found that iNOS expression profoundly increased after knocking down Triad3A (Figure 4H), suggesting that *M. marinum* downregulates iNOS level by upregulating Triad3A (Figure 4H). Collectively, our results demonstrate that *M. marinum* inhibits the expression of iNOS through the Triad3A‐TLR4‐dependent pathway.

### 3.5 Triad3A regulates NO‐mediated antimycobacterial responses via control of TLR4‐NF‐κB

**Figure 5:**
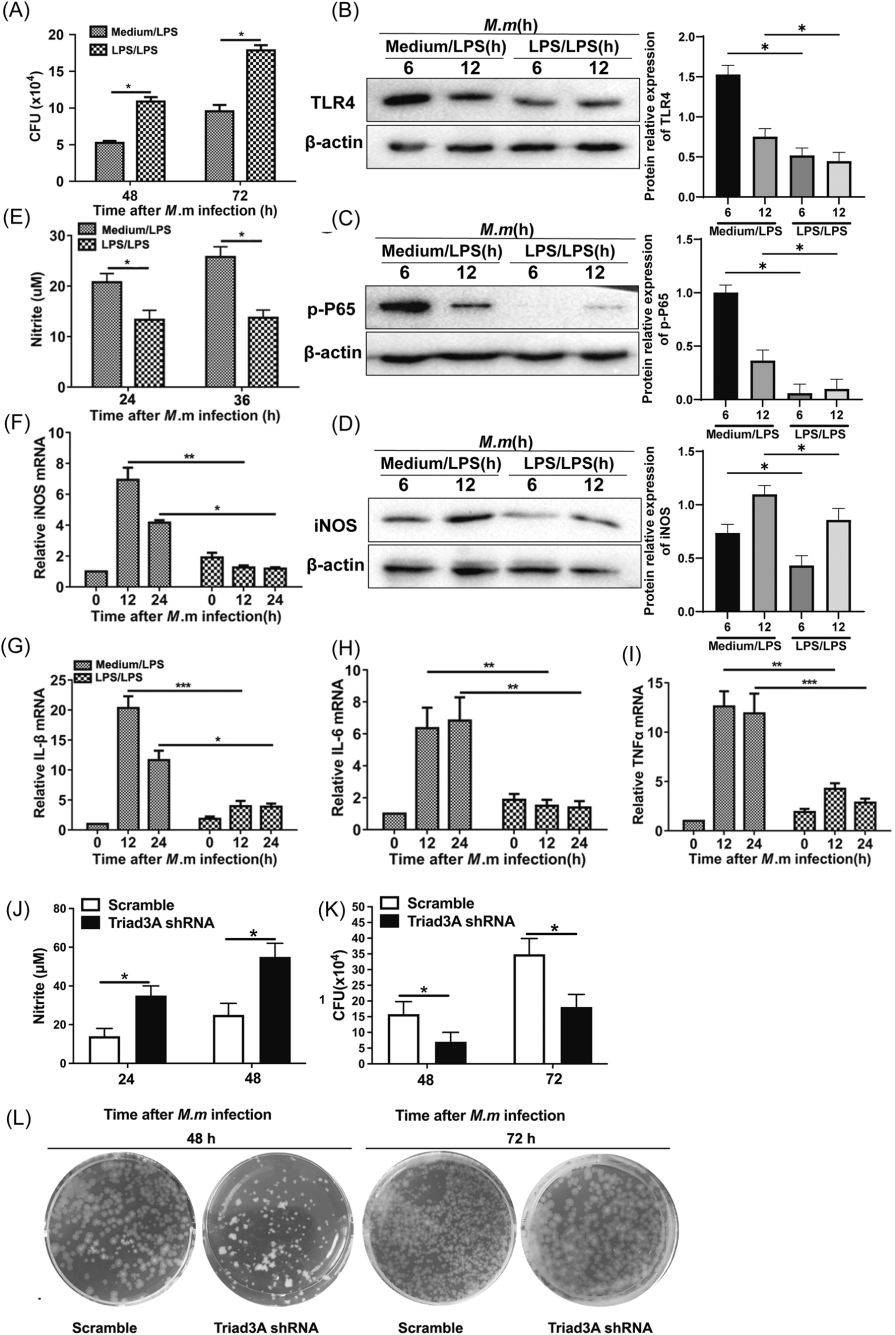
Effect of endotoxin tolerance on antimycobacterial activity and inflammatory response. (A) RAW 264.7 treated with LPS or LPS/LPS were infected with *Mycobacterium marinum*, and intracellular *M. marinum* survival was analyzed by Colony Forming Unit (CFU) assays at the indicated time. (B–D) RAW 264.7 treated with LPS or LPS/LPS were infected with *M. marinum*, the expression of TLR4, p‐P65 and iNOS were detected using Western blot. (E) RAW 264.7 treated with LPS or LPS/LPS were infected with *M. marinum*, *and* supernatant nitrite was determined by the Griess test. (F–I) RAW 264.7 treated with LPS or LPS/LPS were infected with *M. marinum*, iNOS, IL‐1β, IL‐6 and TNF‐α mRNA were detected by qPCR. (J) Stable transfections of Triad3A shRNA and Triad3A Scramble RAW 264.7 were infected with *M. marinum* for 24 and 48 h, and supernatant nitrite was determined by the Griess test. (K and L) Stable transfections of Triad3A shRNA and Triad3A Scramble RAW 264.7 were infected with *M. marinum* at an MOI of 1 for 48 and 72 h. Intracellular *M. marinum* survival was analyzed by CFU at the indicated time. Results represent data from three independent experiments. Two‐way ANOVA test was used to compare differences between indicated the times of inflammatory and tolerance groups. *p < .05, **p < .01. ***p < .001. CFU, colony forming unit.

*M. marinum* infection is associated with diminished TLR4 expression through upregulated Triad3A, and attenuated NF‐κB and iNOS activity. At present, it is yet unclear whether the ability of bacterial clearance during endotoxin tolerance is enhanced or attenuated in a septic host infected persistently with *M. tuberculosis*. Bactericidal activity was assayed by counting the number of colony forming unit (CFU) inside inflammatory and endotoxin tolerant macrophages. We found that macrophages in a tolerant state presented attenuated bactericidal activity compared with inflammatory macrophages (Figure 5A). To clarify the mechanism underlying this different bactericidal activity, we detected the expression levels of TLR4 and iNOS in inflammatory and endotoxin tolerant macrophages infected with *M. marinum*. We found that the expression of TLR4 (Figure 5B), NF‐κB (Figure 5C) and iNOS (Figure 5D) decreased during the endotoxin tolerant state compared to the inflammatory state. Under the endotoxin tolerant condition, the production of NO (Figure 5E) and the mRNA expression of iNOS, IL‐1β, IL‐6, and TNF‐α (Figure 5F–I) were largely suppressed. More importantly, the production of NO could be rescued by silencing the expression of Triad3A (Figure 5J). Correspondingly, the intracellular bacterial burden (displayed using CFU) considerably decreased in cells stably transfected with Triad3A shRNAs rather than scramble shRNAs (Figure 5K–L). These results together strongly suggest that the bactericidal activity of *M. marinum* infected macrophages during endotoxin tolerance was attenuated through suppression of the TLR4‐NFκB‐NO pathway. Inhibition of Triad3A can restore the pro‐inflammatory response phenotype and bacterial clearance ability through this signal pathway.

## 4 DISCUSSION

Tuberculosis sepsis is an infrequent complication of disseminated tuberculosis in HIV‐TB coinfection patients, who require ICU admission. Septic shock can accelerate to *M. tuberculosis* infection. A series of reports showed that ET happened not only in sepsis but also in trauma, surgery, pancreatitis, acute coronary syndrome and cystic fibrosis. An infrequent complication of *M. tuberculosis* infection, serious tuberculosis sepsis, is associated with septic shock with multiple organ dysfunction. It remains unclear, however, whether the bactericidal activity of macrophages is attenuated or not under the state of endotoxin tolerance.

**Figure 6:**
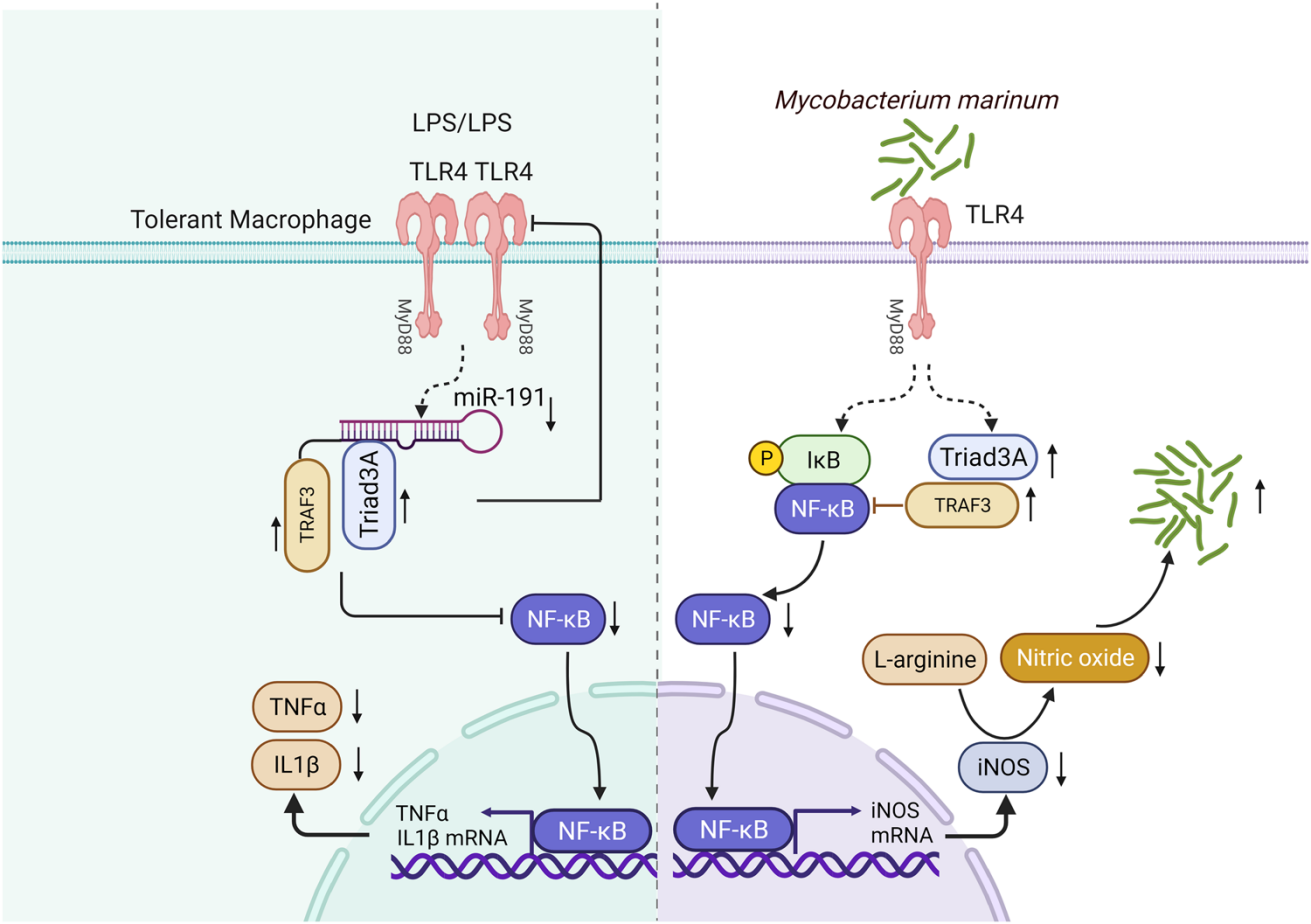
Schematic outline of Triad3A‐regulated endotoxin tolerance and mycobactericidal activity. miR‐191 expression is downregulated in endotoxin‐tolerant macrophages and mediates upregulation of Triad3A and TRAF3 expression, which can further inhibit the expression of TLR4. The expression of Triad3A and TRAF3 is increased in *Mycobacterium marinum* infected macrophages, Triad3A can degrad of TLR4, and TRAF3 negatively regulates NF‐κb, then synergically decreases NF‐κB activity. Thereby, the expression of iNOS and NO is decreased. The downregulation of NO results in the accumulation of *M. marinum* in the macrophage.

In this study, we further demonstrate that Triad3A affects NO production in macrophages under the state of inflammation or endotoxin tolerance (Figure 6). Triad3A is reported to mediate the ubiquitination of TLR4/TLR9 and downregulate the corresponding downstream signaling pathways, causing their degradation and interrupting the NF‐κB signal pathway.^24^, ^51^ Triad3A interacts with TRAF3, ubiquitinated degradation of TRAF3 and further negatively regulates NF‐κB signal. Unlike the downregulation of Triad3A and TRAF3 in LPS‐treated macrophages, studies have shown that Triad3A negatively regulates TRAF3 through Lys48‐linked ubiquitination, knock‐down of Triad3A increased endogenous TRAF3 expression in A549 cells following virus infection.^41^ Our studies displayed that the total TRAF3 and Triad3A protein levels were significantly upregulated in endotoxin‐tolerant macrophages. Triad3A didn't negatively regulate TRAF3 in the endotoxin‐tolerant macrophage. Consistent with these observations, Li and colleagues reported that TRAF3 protein increased in a time‐dependent manner in endotoxin‐tolerant macrophages. Endotoxin tolerance inhibits the degradation of TRAF3 by inhibiting Pellino 1 and K48 ubiquitin.^52^ Our findings are also in line with Wen's results, which displayed that the expression of TRAF3 in the state of ET is upregulated in Kupffer cells to improve the prognosis of sepsis. Degradation of TRAF3 is inhibited during endotoxin tolerance by decreasing the level of TRAF3 K48 ubiquitination.^53^ Similarly, we found that the total Triad3A and TRAF3 protein levels were rapidly upregulated in a time‐dependent manner in *M. marinum*‐infected macrophages. This tendency of Triad3A and TRAF3 is a discrepancy with Nakhaei's findings that Triad3A negatively modulates the RIG‐I‐like receptor signaling by targeting TRAF3 for proteasomal degradation after virus infection.^41^ These results seem to be divergent but not contradictory. Under the state of endotoxin tolerance, Triad3A and TRAF3 are both upregulated due to the regulatory effect of miR‐191, and there is a positive correlation. In the mycobacterial‐infected macrophages, the expression of Triad3A and TRAF3 was downregulated, showing a positive related regulatory effect. These results indicate that in the case of viral or bacterial infection, the intracellular signaling regulatory mechanisms are completely different due to different TLRs that recognize the virus's and bacteria's ligands. TRAF3, as a tri‐faced immune regulator, remained elusive for a long time. In recent years, multiple lines of research have revealed that TRAF3 is a highly versatile regulator that positively regulates interferon production, but negatively controls mitogen activated protein kinase activation (MAPK) and alternative NF‐κB signaling.^54^, ^55^ The different detailed mechanisms by which Triad3A regulates TRAF3 ubiquitination with different domains remain to be clarified in the state of endotoxin tolerance and bacterial infection. A recent study demonstrated that LPS‐tolerant macrophages have significantly decreased cellular respiration.^56^ Other molecules and intracellular pathways may be involved in controlling ET and a series of them require further investigation to verify their potential function in this phenomenon.

Several studies displayed that macrophages treated with repeated LPS show an attenuated pro‐inflammatory reaction. However, few effects on the mycobactericidal activity of NO and its potential mechanism are available in the tolerant macrophage from previous research. Some reports suggested that repeated LPS treatment drastically inhibits bacterial phagocytosis and clearance, leading to severe infection and increased mortality.^57^, ^58^ Other research displayed that macrophages in the state of endotoxin tolerance protect against bacterial infections.^59^, ^60^ The discrepancy may result from time and dose of tolerance, different tolerant animal models, and evaluation method of antibacterial ability, gram‐positive, or gram‐negative bacteria used. Our results further strengthen the assertion that LPS preconditioning in macrophages infected with *M. marinum* reduces pro‐inflammatory cytokines and inflammatory response, attenuates antibacterial activity, and promotes survival of intracellular *Mycobacterium*.

We sought to assess the role of Triad3A in controlling pathogen growth. *M. marinum* has been generally used as a model organism to investigate the tuberculosis pathogenesis of intracellular bacteria and the modulation of cellular immunity.^33^, ^34^ *M. tuberculosis* lipoproteins infection of macrophages induces strong microbicide activity, theoretically triggered by activation of TLRs that reduce the viability of intracellular *Mycobacterium* and inhibit cell‐to‐cell spreading.^61^ In this study, we found that knockdown of Triad3A in macrophages improved antimicrobial activity, which inhibited *Mycobacterium* growth and spread. Our results of Triad3A on mycobactericidal activity are consistent with Xu's findings that overexpression of Triad3A inhibited autophagy in alveolar macrophages through interaction with BECN1, which contributed to *Listeria monocytogenes* growth, while knockdown of Triad3A had the opposite effects.^62^ Autophagy represents an indispensable innate defense and killing bacteria mechanism for eradicating intracellular *Mtb*.^63^ Together, Xu's and our findings demonstrate that downregulation of Triad3A may be a potent weapon against pathogen infection through activating the autophagy mechanism and TLR4‐NO pathway. The actions of the iNOS and the release of NO represent a potent antibacterial mechanism in the host fight against intracellular microorganisms.^64^ Likewise, NO is strictly important in limiting the growth of *M. tuberculosis* and, lacking iNOS in mice, disease progression is fast and fatal.^49^, ^50^, ^61^ Inflammatory responses are mediated by the pro‐inflammatory cytokines, such as IL‐1β and TNF‐α are involved in killing Mycobacterium through different mechanisms.^65^, ^66^ Studies have revealed that complete blockage of the IL‐1 signaling can lead to an increased susceptibility to *M. tuberculosis* infection.^67^ Consistent with these studies, we show here that mRNA expression of iNOS, IL‐1β, IL‐6, and TNF‐α was decreased in the tolerant macrophage infected with *M. marinum*. Meanwhile, mycobacterial loads in cells were decreased in stable Triad3A shRNA cells. Siglec‐E silencing or Neu1 overexpression prevents TLR4 ubiquitination and subsequent degradation by Triad3A during *Leishmania donovani* infection.^68^ The data shown here suggest that the Triad3A‐TLR4 signal is harnessed by mycobacteria or *L. donovani* to inhibit pro‐inflammatory cytokine synthesis and subvert different antibacterial mechanisms of macrophages to provide a compartment for itself.

Studies have reported that transfection of cells with miRNA negatively regulated TLR4‐MyD88 and blocked NF‐κB activity in response to LPS.^69^, ^70^ TLR4‐MyD88 signaling pathway can be negatively modulated by miRNA, attenuate cell viability, augment cell apoptosis, and affect bactericidal activity in macrophages.^71^ Inhibition of miR‐27b resulted in an increased burden of Cryptosporidium infection through indirectly modulating iNOS mRNA stability.^72^ What is beyond our expectation is that intracellular bacterial burden is not affected after transfection of miR‐191 in RAW 264.7 infected with *M. marinum* (data not shown). These results demonstrate that other miRNAs may be involved in bactericidal activity that counteracts or reverses the bactericidal activity mediated by Triad3A. Previous studies have also demonstrated that *M. marinum* infection downregulated miR‐147 and miR‐148 in macrophages, upregulation of miR‐147 and miR‐148 reduced intracellular *M. marinum* survival.^73^

These results together with a comprehension of the susceptibility of certain polymorphic individuals have the potential to direct the exploitation of therapies targeting specific infections. The next research will show the increasing relevance of Triad3A‐TLR signaling to clinical practice. Gene polymorphisms of TLRs and components of this signaling molecule have been displayed to influence the severity of sepsis and susceptibility to aggressive bacterial diseases.^74^, ^75^, ^76^, ^77^ Single nucleotide changes increased susceptibility to numerous major infectious diseases in heterozygous individuals.^78^ These findings are consistent with previous studies that an excessive inflammatory reaction may render patients more susceptible to developing severe bacterial malaria infection disease.^79^, ^80^ Host‐directed therapy is an effective approach that enhances protective immune responses against the pathogen, and reduces exacerbated infection and inflammation.^81^, ^82^, ^83^, ^84^ We have clarified the effect of Triad3A on TLR4 regulation and mycobactericidal activity in the state of sepsis. Increased clarification of the pro‐inflammatory and tolerant phases of the septic response may help patent antimycobacterial infection and immunostimulatory therapies to be used properly in the host.

In summary, we found Triad3A could potently attenuate antimycobacterial activity by inhibiting NF‐κB‐induced NO production via TLR4. Given the importance of NO in the innate immune responses during mycobacterial infection, this is the first study to identify a potent function of Triad3A through degradation of TLR4, which further inhibits the expression of iNOS and NO generation. This phenomenon can be considered an escape mechanism for mycobacterium and facilitating its survival and proliferation in the macrophage. Meanwhile, this represents a potential target for drug development in the governance of sepsis accompanied with tuberculosis.

## AUTHOR CONTRIBUTIONS

**Yongwei Qin**: Designed; writing—original draft. **Jinliang Chen**: Investigated. **Kuang Xu**: Investigated. **Yang Lu**: Collected experimental materials and analyzed data. **Feifan Xu**: Revised the draft; funding application; administrative support. **Jiahai Shi**: Revised the draft; funding application; administrative support. All the authors approved the final version.

## CONFLICT OF INTEREST STATEMENT

The authors declare no conflict of interest.

## ETHICS STATEMENT

All animal experiments had gained approval from the Medical Ethics Committee of Nantong University and were carried out in accordance with the Guidelines for the Care and Use of Animals for Scientific Research.

## Data Availability

Data in this study is available from the corresponding author on reasonable request.
